# Scutellarin Prevents Angiogenesis in Diabetic Retinopathy by Downregulating VEGF/ERK/FAK/Src Pathway Signaling

**DOI:** 10.1155/2019/4875421

**Published:** 2019-12-28

**Authors:** Lingli Long, Yubin Li, Shuang Yu, Xiang Li, Yue Hu, Tengfei Long, Liqin Wang, Wenwen Li, Xiaoxin Ye, Zunfu Ke, Haipeng Xiao

**Affiliations:** ^1^Department of Endocrinology, The First Affiliated Hospital, Sun Yat-sen University, Guangzhou 510080, China; ^2^Translation Medicine Center, The First Affiliated Hospital, Sun Yat-sen University, Guangzhou 510080, China; ^3^The Reproductive Center, The First Affiliated Hospital, Sun Yat-sen University, Guangzhou 510080, China; ^4^Department of Spine Surgery, The First Affiliated Hospital, Sun Yat-sen University, Guangzhou 510080, China; ^5^Department of Gynaecology and Obstetrics, Sun Yat-sen Memorial Hospital, Sun Yat-sen University, Guangzhou 510120, China; ^6^Department of Radiology, The First Affiliated Hospital, Sun Yat-sen University, Guangzhou 510080, China; ^7^Laboratory Animal Center, The First Affiliated Hospital, Sun Yat-sen University, Guangzhou 510080, China; ^8^University of New South Wales, Sydney, High St. Kensington, NSW, Australia; ^9^Department of Pathology, The First Affiliated Hospital, Sun Yat-sen University, Guangzhou 510080, China

## Abstract

**Background:**

Diabetic retinopathy (DR) is a serious microvascular complication of diabetes. This study demonstrates the antiangiogenic effects of scutellarin (SCU) on high glucose- and hypoxia-stimulated human retinal endothelial cells (HRECs) and on a diabetic rat model by oral administration. The antiangiogenic mechanisms of SCU *in vitro* and *in vivo* were investigated.

**Method:**

HRECs were cultured in high glucose- (30 mM D-glucose) and hypoxia (cobalt chloride-treated)-stimulated diabetic condition to evaluate the antiangiogenic effects of SCU by CCK-8 test, cell migration experiment (wound healing and transwell), and tube formation experiment. A streptozotocin-induced type II diabetic rat model was established to measure the effects of oral administration of SCU on protecting retinal microvascular dysfunction by Doppler waveforms and HE staining. We further used western blot, luciferase reporter assay, and immunofluorescence staining to study the antiangiogenic mechanism of SCU. The protein levels of phospho-ERK, phospho-FAK, phospho-Src, VEGF, and PEDF were examined in HRECs and retina of diabetic rats.

**Result:**

Our results indicated that SCU attenuated diabetes-induced HREC proliferation, migration, and tube formation and decreased neovascularization and resistive index in the retina of diabetic rats by oral administration. SCU suppressed the crosstalk of phospho-ERK, phospho-FAK, phospho-Src, and VEGF *in vivo* and *in vitro*.

**Conclusions:**

These results suggested that SCU can be an oral drug to alleviate microvascular dysfunction of DR and exerts its antiangiogenic effects by inhibiting the expression of the crosstalk of VEGF, p-ERK, p-FAK, and p-Src.

## 1. Introduction

Diabetic retinopathy (DR), an important cause of permanent vision loss in working-age adults, is a serious microvascular complication of diabetes [1]. The pathogenesis of DR is complicated because multiple factors induce the disease. Recent studies have revealed that the retinas of diabetic patients quickly react to hyperglycemia and hypoxia, resulting in imbalances between proangiogenic and antiangiogenic processes [2]. Hyperglycemia leads to retinal capillary destruction, pericyte apoptosis, and extreme vasoconstriction, all of which are related to the elevated secretion of angiogenic factors in DR. Many vasoregulatory factors have been proven to be involved in the pathogenesis of retinal neovascularization (NV). Vascular endothelial growth factor (VEGF) is one of the most important angiogenic factors involved in the vascular pathology of DR [3]. In the retina, VEGF is expressed in many types of cells, such as retinal endothelial cells, retinal pigment epithelium (RPE) cells, Müller cells, astrocytes, and ganglion cells [3]. Among these, retinal endothelial cells are known to respond quickly during the pathogenesis of DR to form vessels [4]. Overexpression of VEGF is a major cause of retinal neovascularization and vascular leakage. VEGF promotes migration, proliferation, and tube formation in human retinal endothelial cells (HRECs) through autocrine signaling, and it also regulates the cooperation of other cells during angiogenesis through paracrine signaling in the retina [5]. Thus, VEGF has been considered as a therapeutic target for antiangiogenesis in diabetic retinopathy [5, 6]. The most popular treatment for DR includes laser surgery and intraocular injection of VEGF antagonists, but these therapies can result in retinal neuron destruction, irreversible retinal damage, and even loss of vision [7, 8]. Therefore, development of an effective oral drug with fewer detrimental effects would be a great improvement for DR therapy.

Scutellarin (SCU, S1.), a known flavone glycoside, is the primary active ingredient in breviscapine, which is a mixture of flavonoid glycosides extracted from the Chinese herb Erigeron breviscapus (vant.) Hand. Mazz [9, 10]. SCU exhibits a variety of pharmacological properties, including antioxidative [11] and anti-inflammatory [12–15] properties, and it can also correct vascular dysfunction by reducing blood viscosity and improving microcirculation. Chen et al. have demonstrated that SCU can significantly reverse cardiac structural disorganization and ameliorate cardiac dysfunction in diabetic cardiomyopathy via the PKC/NF-*κ*B/TNF-*α*/c-fos signal transduction pathway [16]. Actually, SCU has been extensively used in clinics to treat conditions associated with vascular dysfunction, such as cardiovascular and cerebrovascular injury [17–20]. Our previous study indicated that SCU could effectively reverse testicular microcirculatory dysfunction by controlling reactive oxygen species (ROS)/Bcl-2/Bax and the ROS/microcirculation/staving pathway in diabetic rats. We also found the efficacy of orally delivered SCU is excellent in diabetic rats [21].

On this basis, the main objectives of the current study were to examine whether SCU exerts beneficial effects against angiogenesis and microvascular abnormalities in *in vitro* and *in vivo* diabetic model. In addition, we also evaluated the SCU-triggered signaling pathway involved in ameliorating neovascularization in DR.

## 2. Materials and Methods

### 2.1. Reagents and Antibodies

Scutellarin was purchased from Sichuan Jiexiang Pharmaceutical Industry Ltd. (Sichuan, China) and was prepared based on manufacturer's instruction for cellular experiments. In *in vivo* experiments, SCU was formulated in ddH_2_O and administered *via* oral gavage at 40 mg/kg. The SCU is shown in S1. CoCl_2_ and glucose were purchased from Guangzhou Huaqisheng Industry Ltd. (Guangzhou, China, AR). Primary antibodies against GAPDH, AKT, p-Akt, ERK1/2, p-ERK1/2, FAK, p-FAK, Src, and p-Src and horseradish peroxidase- (HRP-) conjugated secondary antibodies were obtained from Cell Signaling Technology (Beverly, MA, USA).

### 2.2. Cell Culture and Culture Conditions

HRECs were bought from Shanghai Bioleaf Biotech Co., Ltd. (Shanghai, China). HRECs were plated on a T25 culture flask containing ECM (Gibco BRL, Grand Island, NY, USA), 10% FBS (Gibco BRL, Grand Island, NY, USA), 100 U/ml penicillin, and 100 U/ml streptomycin. All cell cultures were maintained at 37°C in a 5% CO_2_ incubator. 30 mM D-glucose and 200 *μ*m/l cobalt chloride (CoCl_2_) were added into the HRECs to imitate the hyperglycemia and hypoxic environment of diabetes. According to previous studies, CoCl_2_ as a chemically hypoxic mimetic has been used to induce hypoxia in cell lines [22, 23].

### 2.3. Transfection

The small interfering RNA (siRNA) duplexes were predesigned with the online software (Stealth RNAi Pre-Designed siRNAs) provided by Ambion (Thermo Scientific™) (http://www.thermofisher.com/cn/zh/home/life-science/rnai/synthetic-rnai-analysis/stealth-select-rnai.html) and constructed by Gene Pharma (Gene Pharma Co., Suzhou, China). The siRNAs had been verified to be efficient before all experiments. Cells were plated at a concentration of 1 × 10^5^ cells/well in 6-well plates and transduced with the small interfering RNA (siRNA) using lipofectamine RNAiMAX transfection reagent (Invitrogen, USA) according to the manufacturer's instructions. Different amounts of 20 *μ*M siRNA duplexes were mixed with 5 *μ*l/well of transfection reagent and Opti-MEM reduced serum medium (Invitrogen, USA) to total volume of 500 *μ*l and incubated for 20 min. The mixture was applied to the cell 16 hours at 37°C in 5% CO_2_.

### 2.4. Glucose and Triglyceride Content Measurement

The same protein amount from HREC lysates from each group was used to measure the internal glucose and triglyceride content. The Reflotron Plus/Sprint System (Roche) was used to measure the *δ*-D-gluconolactone and hydrogen peroxide. Colorimetric assay was used to quantify triglyceride. First, glycerol-3-phosphate was obtained from the glycerol in the triglycerides and then degraded to hydrogen peroxide. Secondly, the SPINREACT system was used to spectrophotometrically determine quinone at 505 nm which was a product of enzymatic reactions of hydrogen peroxide.

### 2.5. Glucose Uptake Assay

The 2-Deoxy-D-glucose (2DG) Uptake Measurement Kit (Cosmo Bio CSR-OKP-PMG-K01E) was used to measure glucose uptake, following the steps in the previous study [24].

### 2.6. Cell Viability Assay

HRECs were seeded in 96-well plates in 200 *μ*l DMEM containing 10% FBS and cultured at 37°C for 24 h. After incubation for 0 h, 24 h, and 48 h, 10 *μ*l of CCK-8 (CCK-8; Dojindo Molecular Technologies Inc., Kumamoto, Japan) reagent was added to each well. The cells were incubated for an additional 2 h, and the absorbance values of samples were measured at 570 nm.

### 2.7. Migration Assay

#### 2.7.1. Wound Healing Assay

All experiments were performed in the presence of 5 *μ*g/ml of mitomycin C to inhibit cell proliferation. HRECs were cultured in ECM containing 0.5% FBS treated with 5 *μ*g/ml mitomycin C [25] and with or without 30 mM of glucose, 200 *μ*m CoCl_2_, and 10 *μ*m/l SCU for 24 h. HRECs were cultured in 5 *μ*g/ml of mitomycin C and 15 mM mannitose as osmotic control. 5 *μ*g/ml mitomycin C completely inhibits cell proliferation but does not reduce cell numbers. Thus, 5 *μ*g/ml mitomycin C was used to impede cell proliferation and perform wound healing assays on all groups in the presence of 5 *μ*g/ml mitomycin C.

After 0 and 24 h incubation, the cells were rinsed with PBS and wound closure was monitored under an inverted phase contrast microscope; randomly chosen fields were photographed under a light microscope (model IX71; Olympus, Tokyo, Japan) at ×100 magnification. For quantitative assessment, the wound area was determined by Image-ProPlus software 5.1 (Media Cybernetics, Inc. Silver Spring, MD, USA). Each experiment was done in triplicate.

#### 2.7.2. Cell Migration Abilities by Transwell Assay

A volume of 200 *μ*l HRECs was taken and placed in a transwell chamber (0.4 *μ*m, Corning, USA) containing 500 *μ*l 20% serum medium for cultivation. HRECs in 15 mM mannitol were cultured as osmotic control. After 24 h of cultivation, the medium was sucked, and the noninvasive cells in the upper chamber were removed, invasive cells embedded in the membrane of the transwell fixed with 4% paraformaldehyde, stained with crystal violet for 15 min.

The cells were observed under a microscope and recorded. To quantify the number of migratory cells, HRECs with the crystal violet staining were eluted with glacial acetic acid, and the UV absorption of crystal violet was measured at 450 nm by a microplate reader (ELX50, BIO-TECH, China).

### 2.8. Matrigel Angiogenesis Assay

The angiogenic ability of HRECs was measured by seeding 10000 cells (in 10 *μ*l basal medium) onto 10 *μ*l solidified Matrigel (ECMatrix, Millipore) in an m-Slide Angiogenesis (Ibidi, Martinsried, Germany). 50 *μ*l of test medium (with/without glucose, CoCl_2_, and SCU) was added and was incubated at 37°C for 24 h. HRECs in 15 mM mannitol were cultured as osmotic control. Images were recorded using an Olympus microscope (IX71) and analyzed by AngioSys 1.0 (TCS Cellworks, Buckingham, England). The number of tubules, junctions, and total tubule length (relative to control medium) was viewed as a measure for angiogenesis.

### 2.9. Western Blot Analysis

To understand the molecular mechanism of action of SCU, cells or retinas from rats were lysed with RIPA Lysis Buffer (Santa Cruz Biotechnology, Santa Cruz, CA) containing protease inhibitors (Complete; Roche, Mannheim, Germany) and phosphatase inhibitors (PhosStop; Roche, Mannheim, Germany). The protein concentration was determined using the BCA assay (Beyotime Biotechnology, Haimen, China) and equalized before loading. 50 *μ*g of proteins extracted from cultured cells or tissues was separated by SDS-PAGE and transferred onto PVDF membranes. Blots were blocked with 5% nonfat dried milk for 1 h and then incubated overnight at 4°C with the relevant primary antibody. HRP-conjugated secondary antibodies were detected by enhanced chemiluminescence reagent (Millipore Corp., Bedford, MA). GAPDH protein was used to normalize the total tissue lysate on the same membrane. All antibody dilutions were done with 1 : 1000, except for the PEDF antibody, which was used at 1 : 500 dilutions.

### 2.10. Immunofluorescence Analysis

HRECs were fixed in 4% PFA for 30 minutes and permeabilized with 0.3% Triton X-100 for 30 minutes. Blocking was performed with 5% normal goat serum for 1 hour. The cells were incubated overnight at 4°C in the primary antibodies (anti-VEGF antibody, 1 : 200). After washing three times in PBS, the primary antibodies were probed with the secondary antibodies Alexa Fluor 594 goat anti-rabbit (1 : 500, Invitrogen, Camarillo, CA) and Alexa Fluor 594 goat anti-mouse (1 : 500, Invitrogen) for 1 h at room temperature. Finally, the coverslips were washed in PBS three times and mounted using ProLong Gold Antifade Reagent containing 4′-6-diamidino-2-phenylindole (DAPI) (Molecular Probes, Invitrogen). Image was analyzed by MetaMorph Imaging Software (Molecular Devices). VEGF intensity (arbitrary fluorescence units of red) was quantified, integrated, and normalized to the integrated DAPI intensity (equivalent to cell number). Results were showed as the ratio of arbitrary fluorescence units to integrated DAPI intensity.

### 2.11. Luciferase Reporter Assay

VEGF promoter reporter construct was generated by polymerase chain reaction. Primers with unique restriction site Mlu1 and XhoI sequences were used to amplify selected regions containing the fragment of 2000 bp VEGF promoter using genomic mouse DNA as a template. Cells were plated at a density of 1 × 10^4^ cells/cm^2^ and transfected for 4 hours with a luciferase reporter construct plasmid mixture of 2.9 mg of the luciferase promoter reporter and 0.1 mg of the pGL4.74 Renilla luciferase control vector (Promega, Madison, WI, USA) according to the manufacturer's instructions. Luciferase activity was measured using the luciferase assay kit (Promega) according to the manufacturer's instructions. The data were normalized to Renilla luciferase activity, and relative luciferase units (RLU) were calculated as firefly luciferase activity/Renilla luciferase activity.

### 2.12. Animals

The rats were maintained in the Center for Experimental Animals at Sun Yat-sen University. The experiments outlined in this article conform to the National Institutes of Health Guide for the Care and Use of Laboratory Animals. All experimental protocols and animal handling procedures were approved by the Sun Yat-sen University Committee on Animal Care. Male Sprague-Dawley rats weighing 230-250 g were obtained from the Experimental Animal Center of Sun Yat-sen University. Rats were randomly assigned to 4 groups (*n* = 8/group): [1] control group, normal rats without SCU treatment; [2] SCU group, normal rats with SCU treatment; [3] DR group, type 2 diabetes mellitus (T2DM) rats without SCU treatment; and [4] DR+SCU group, T2DM rats with SCU treatment. Control and SCU rats were fed for 8 weeks on normal standard diets, while T2DM rats were fed a high-fat diet for 8 weeks, comprising 30% calories from fat, 15% from protein, and 55% from carbohydrate. After 8 weeks, rats in the T2DM groups were fasted overnight and given a single intraperitoneal injection of streptozotocin (STZ, Sigma, USA) diluted in citrate buffer 0.1 mol/l (pH 4.0) at a dose of 30 mg/kg the following morning, and the high-fat diet feeding was continued. Rats in the control and SCU groups were injected with citrate buffer. Blood glucose and weights were monitored 3 days after the STZ or citrate buffer injection. The diabetic state was confirmed by measuring the tail blood glucose (BG) level at 7 days after STZ injection. Rats whose blood glucose levels were >16.7 mmol/l on at least three occasions were deemed to be diabetic [26, 27]. T2DM rats were randomly assigned to the DR group and the DR+SCU group. After that, control and DR group rats were intragastrically administrated with phosphate buffer (0.1 M, pH 7.4) for 8 weeks, while the SCU group and the DR+SCU group were intragastrically administrated with SCU (40 mg/kg/day) for 8 weeks. Eight weeks after STZ injection, all rats were anesthetized by luminal injection and sacrificed.

### 2.13. Color Doppler Ultrasound

Before sacrifice, color Doppler ultrasound vascular analysis of the rat's eye was performed as previously described [28]. Briefly, following anesthesia, MS 400 probe (18–38 MHz) color Doppler sonography (VisualSonics Vevo 2100, Toronto, Canada) was used in our experiments to scan the central retinal vasculature. The arterial tracings of Doppler spectral analysis were applied to record Doppler waveforms and color images were recorded in real time. The resistivity index (RI) of the central retinal artery was calculated by the formula

(1) $$\begin{array}{c} \text{RI}=\frac{\text{SV}-\text{DV}}{\text{SV}}, \end{array}$$

where DV is the diastolic velocity and SV is the peak systolic velocity.

An increased central retinal artery RI value is indicative of vasoconstriction at the capillary or precapillary level within the retina.

### 2.14. Histopathology Examination

For histopathological analysis, the rats were killed with an overdose of chloral hydrate. The eyes were enucleated and fixed in 10% buffered formalin solution, dehydrated, embedded in paraffin, and then sliced into 5 *μ*m thick sections. After being deparaffinized, hematoxylin and eosin-stained slides were prepared by using the standard method. Ten histological sections from each eye (*n* = 5) were examined, and the histological severity of the retina was graded semiquantitatively in a blinded fashion by two pathologists (Xiaofang Lu and Canqiao Luo) on a scale of 1 (no disease) to 5 (maximum disease).

### 2.15. Statistical Analysis

Data were expressed as mean ± SD. All statistical analyses were performed using SPSS 17.0 software (SPSS, Armonk, NY, USA). One-way ANOVA and Student's *t*-test were used to compare the differences, which was performed with the Prism 7.0 software (GraphPad, San Diego, CA, USA). A value of *P* < 0.05 was considered statistically significant.

## 3. Results

### 3.1. Glucose/Cobalt Chloride- (CoCl_2_-) Treated HRECs Showed Increased Glucose Content, Glucose Uptake, and Triglyceride Accumulation

**Figure 1:**
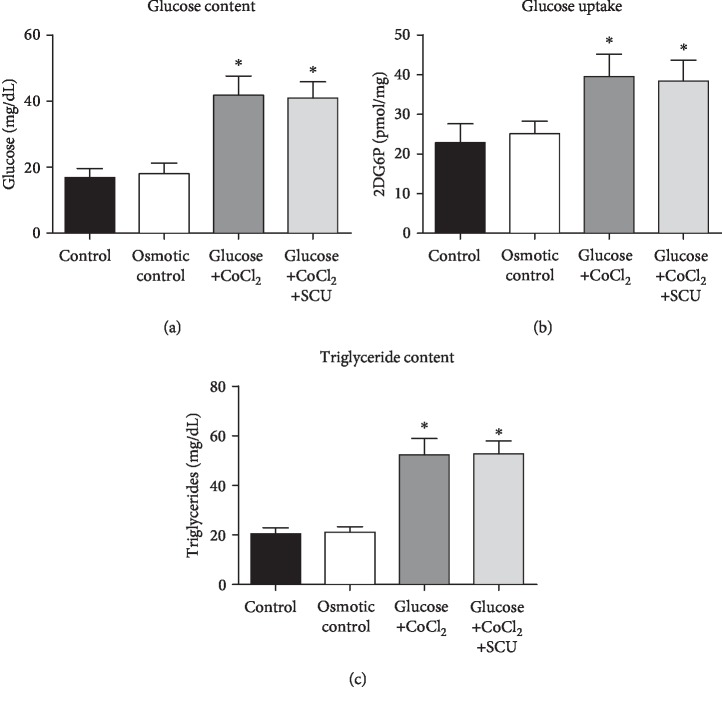
Glucose content, glucose uptake, and triglyceride content in HRECs. HRECs were cultured in control medium, mannitol-supplemented medium (osmotic control), glucose/CoCl_2_-supplemented medium, and glucose/CoCl_2_+SCU-supplemented medium, and (a) glucose content, (b) glucose uptake, or (c) triglyceride content was measured. Values are expressed as median ± SD (*n* = 5). ^∗^*P* < 0.05 compared to the control group and the osmotic group.

DR environmental properties not only include hyperglycemia but also retinal hypoxia. High glucose and CoCl_2_ have been used to mimic diabetes-induced hyperglycemia and hypoxia in many studies [29–32]. Therefore, D-glucose and CoCl_2_ have been used to imitate the hyperglycemia/hypoxia DR environment in HRECs of our study. To prove that a hyperglycemia/hypoxia HREC model was successfully established by treatment with D-glucose (30 mM) and CoCl_2_ (200 *μ*m/l), glucose content, glucose uptake, and triglyceride content were measured in HRECs that were cultured in control medium (control group), mannitol-supplemented medium (osmotic control group), glucose/CoCl_2_-supplemented medium (glucose+CoCl_2_ group), and glucose/CoCl_2_+SCU-supplemented medium (glucose+CoCl_2_+SCU group). The results indicated that the glucose content, glucose uptake, and triglyceride content of the HRECs in the glucose/CoCl_2_ group were higher than those in the control and osmotic control groups (Figures 1(a)–1(c)), which indicated that HRECs treated with D-glucose (30 mM) and CoCl_2_ (200 *μ*m/l) successfully established a diabetic cell model *in vitro*. On the other hand, there is no significant difference in glucose content, glucose uptake, and triglyceride content between the glucose/CoCl_2_ group and the glucose/CoCl_2_+SCU group, which indicated that SCU seldom affects glycolipid metabolism in HRECs under high glucose/hypoxia condition.

### 3.2. SCU Inhibited HREC Proliferation, Migration, and Tube Formation Induced by Hyperglycemia and Hypoxia

**Figure 2:**
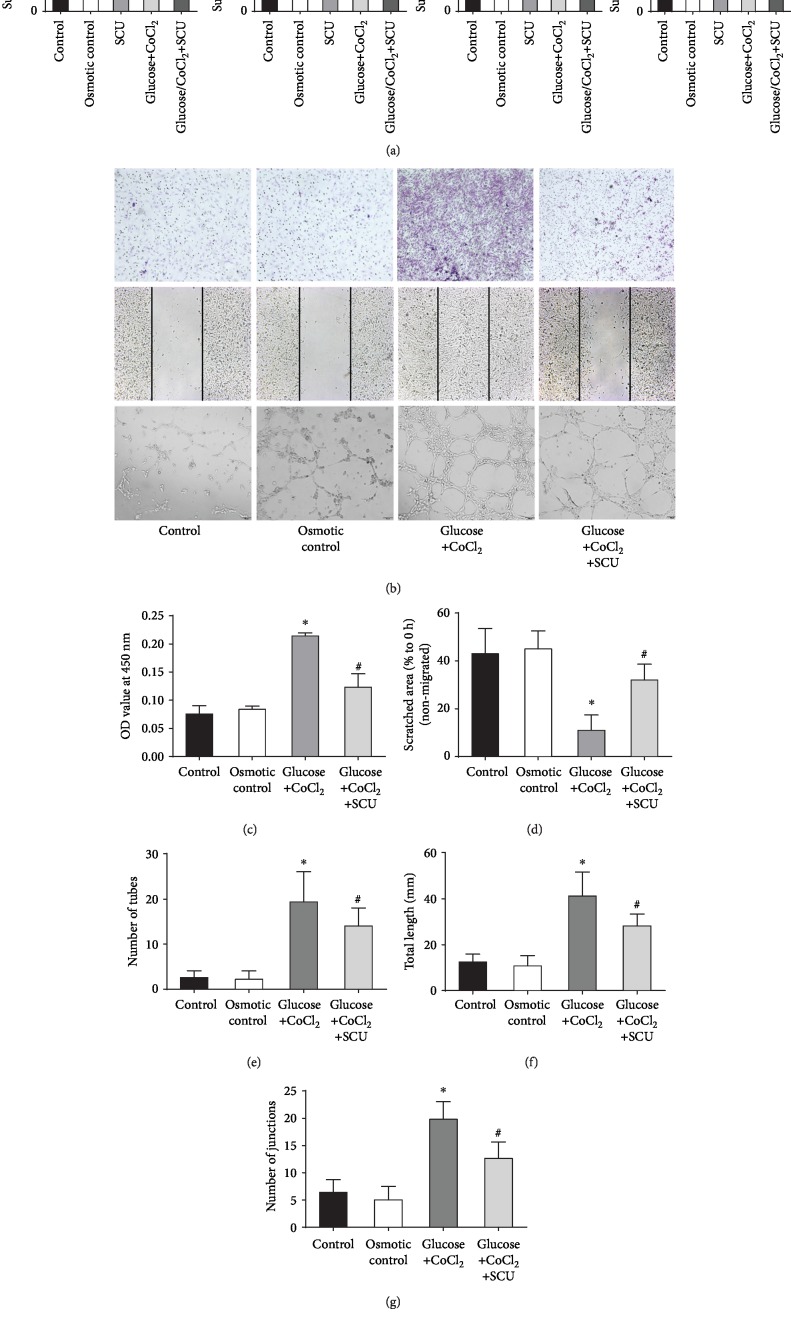
SCU inhibited glucose/CoCl2-induced HREC proliferation, migration, and tube formation. (a) HRECs were treated with SCU (10 *μ*m/l), glucose (30 mm/l), and CoCl_2_ (200 *μ*m/l) as the graph showed for 0 h, 24 h, 48 h, or 72 h, and cell survival rate was determined using the CCK-8 assay. Cell proliferation was calculated as a percentage of the control. Data are presented as mean ± SD (*n* = 3). ^∗^*P* < 0.05 compared to the control group, the osmotic control group, the SCU group, and the SCU+glucose/CoCl_2_ group. ^#^*P* < 0.05 compared to the glucose+CoCl_2_ group. The control, osmotic control, and SCU groups have no statistical significance (*P* > 0.05). (b) The results of transwell assay, wound healing assay, and Matrigel tubulogenesis assay. Scale bar = 50 *μ*m. (c) The quantitative results of HRECs migrated of transwell assay is measured by a microplate reader. (d) Scratched areas, which were not covered by migrated cells, were measured using ImageJ (NIH) for the quantification. (e–g) The images of Matrigel tubulogenesis assay were quantified as the number of junctions, number of tubules, and total tubule length. Images were processed using Adobe Photoshop CS2 9.0.2 (Adobe Systems, San Jose, CA) with the Image Processing Tool Kit plug-ins (Reindeer Graphics, Asheville, NC) and analyzed using AngioSys 1.0 (TCS Cellworks, Buckingham, England). All the data in (c)–(g) were presented as mean ± SD; ^∗^*P* < 0.05 compared to the control group, the osmotic control group, and the SCU+glucose+CoCl_2_ group; ^#^*P* < 0.05 compared to the control group, the osmotic control group, and the glucose+CoCl_2_ group.

**Figure 3:**
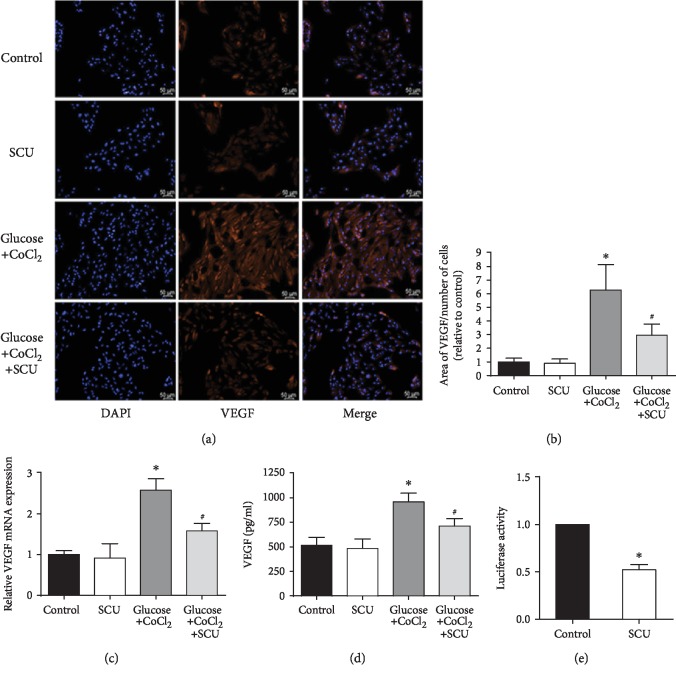
SCU attenuated the high expression of VEGF induced by glucose and CoCl_2_ in HRECs. (a) Representative microscopic images of immunofluorescent staining for VEGF in HRECs of all groups (scale bar = 50 *μ*m). (b) VEGF intensity compared with DAPI intensity (relative to control) was quantified. (c) mRNA expression level of VEGF in HRECs of all groups is shown. (d) Excretive VEGF in the control medium and the conditional medium was measured by ELISA. (e) VEGF promotes transcriptional activities of HRECs treated with SCU. Data are presented as mean ± SD (*n* = 3); ^∗^*P* < 0.05 compared to the control group or the SCU group; ^#^*P* < 0.05 compared to the glucose+CoCl_2_ group, the SCU group, and the control group.

To assess the influences of SCU on HRECs under hyperglycemic and hypoxic conditions, we evaluated the survival rates of HRECs in the control group, the osmotic control group, the SCU group, the glucose+CoCl_2_ group, and the SCU+glucose/CoCl_2_ group at 0 h, 24 h, 48 h, and 72 h by CCK-8 assay (Figure 2(a)). The results showed that HRECs in the glucose+CoCl_2_ condition had increased proliferation at 48 h and 72 h but this effect was reversed by treatment with SCU (10 *μ*m/l). Thus, SCU exerted an antiproliferative effect on HRECs under diabetic conditions. Transwell migration assays and wound healing assays were performed to measure the ameliorative effects of SCU on increased HREC motility under diabetic conditions (Figure 2(b)). In the transwell experiment, we observed increased migration in HRECs cultured with glucose+CoCl_2_. In contrast, in the glucose+CoCl_2_+SCU group, HREC migration was significantly decreased. Similarly, the wound healing assay indicated that glucose/CoCl_2_ enhanced the migration of HRECs treated with mitomycin C to exclude the influence of proliferation. Thus, we also found that SCU decreases the migratory ability of HRECs under glucose+CoCl_2_ treatment (Figures 2(b)–2(d)). Together, the results of these two experiments indicate that SCU can decrease the migration of HRECs under diabetic conditions. Matrigel angiogenesis assays were further used to assess the ability of HRECs to form tubes, an important step of angiogenesis. The Matrigel tube formation assay (Figures 3(b)–3(g)) demonstrated that the number of tubes, tube length, and number of branch points were markedly higher in the glucose+CoCl_2_ group than in the control group and the osmotic control group, but these parameters were significantly decreased with SCU treatment. Therefore, we suggest that SCU can decrease tube formation by HRECs under diabetic conditions. Importantly, HREC proliferation, migration, and tube formation were not significantly different between the osmotic control group and the negative control group, demonstrating that the alterations in the glucose+CoCl_2_ group were unrelated to osmotic stress. Proliferation, migration, and tube formation in HRECs are known to be important indicators of angiogenesis. In summary, these results provide compelling evidence that SCU effectively reduces angiogenesis in HRECs under hyperglycemic and hypoxic conditions, suggesting promising antiangiogenic effects of SCU in DR.

### 3.3. SCU Attenuated Diabetes-Induced VEGF, p-ERK, p-FAK, and p-Src in HRECs

**Figure 4:**
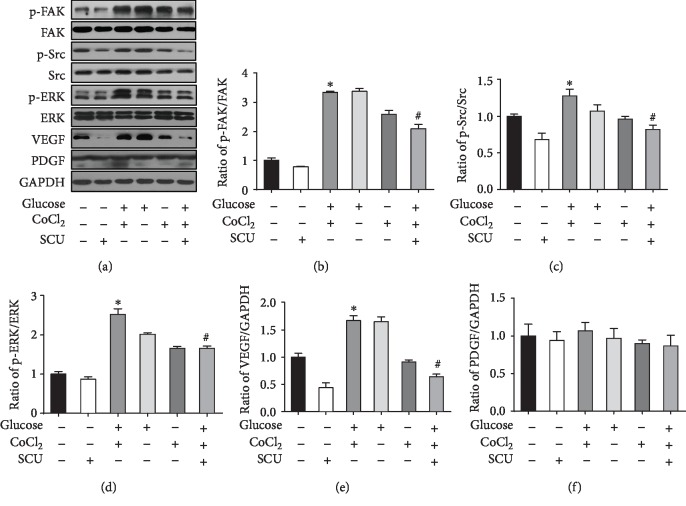
SCU decreased the activation of p-ERK, p-FAK, p-Src, and VEGF, but not PEDF of HRECs under diabetic condition. (a) Western blot analysis was used to determine the protein expression of VEGF, PEDF, FAK, p-FAK, p-Src, Src, p-ERK, ERK, and p-ERK in HRECs with different treatments. GAPDH was used as loading control. Data are shown as mean ± SD and are presented as ratio (b–f); ^∗^*P* < 0.05 compared to the control group; ^#^*P* < 0.05 compared to the control group and the glucose+CoCl_2_ group.

VEGF is known to play an important role in regulating endothelial metabolism, especially under diabetic conditions, and can increase proliferation, migration, and tube formation in endothelial cells [33]. Based on the results in Figure 2, we propose that the increasing angiogenesis ability of HRECs was related to the high expression of VEGF induced by hypoglycemia and hypoxia. We thus evaluated the distribution of VEGF in HRECs by immunofluorescence microscopy and found increased VEGF in the glucose+CoCl_2_ group that was attenuated by the treatment with SCU (Figure 3(a)), based on quantification of VEGF intensity/DAPI intensity (Figure 3(b)). The cytoplasm and excretory VEGF were increased after the stimulation of high glucose and hypoxia, and SCU showed an ability to decrease the glucose/hypoxia-induced VEGF (Figures 3(c) and 3(d)). In order to prove that SCU directly regulates the transcription of VEGF, luciferase reporter assay was performed. Our results showed that the transcriptional activity of VEGF in HRECs was markedly decreased by SCU. Furthermore, our results also indicated that the phosphorylation of p-ERK, p-Src, and p-FAK was inhibited in HRECs with SCU treatment, but the expression of pigment epithelium-derived factor (PEDF), an antiangiogenic factor, was seldom affected (Figure 4).

### 3.4. SCU Attenuates Diabetes-Induced Angiogenesis in HRECs by Impeding Crosstalk of VEGF, p-ERK, p-Src, and p-FAK

**Figure 5:**
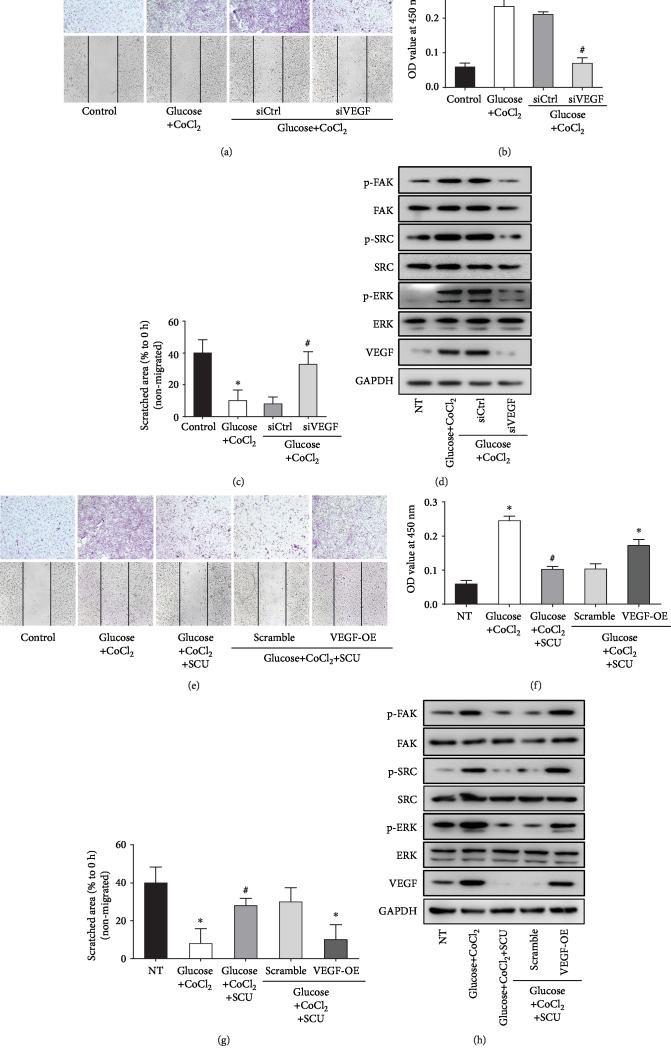
SCU targeted VEGF to attenuate hyperglycemia-induced tubulogenesis. (a) The migration of HRECs and HRECs^kd-VEGF^ treated with glucose+CoCl_2_ was measured by transwell migration assays and wound healing assays. (b) The quantitative results of HREC transwell experiment were measured by a microplate reader. (c) Scratched areas, which were not covered by migrated cells, were measured using ImageJ (NIH) for the quantification. Data in (b) and (c) were presented as mean ± SD (*n* = 5); ^∗^*P* < 0.05 compared to the control group and the glucose+CoCl_2_+siVEGF group. ^#^*P* < 0.05 compared to the glucose+CoCl_2_ group and the glucose+CoCl_2_+siControl group. (d) Western blot analysis of p-FAK, p-Src, and p-ERK in HRECs and HRECs^kd-VEGF^ treated with glucose+CoCl_2_. (e) The migration of HRECs and HRECs^OE-VEGF^ treated with glucose+CoCl_2_+SCU was measured by transwell migration assays and wound healing assays. (f) The quantitative results of HREC transwell experiment were measured by a microplate reader. (g) Scratched areas, which were not covered by migrated cells, were measured using ImageJ (NIH) for the quantification. Data in (f) and (g) were presented as mean ± SD (*n* = 5); ^∗^*P* < 0.05 compared to the control group, the glucose+CoCl_2_+SCU group, and the glucose+CoCl_2_+SCU+scramble group. ^#^*P* < 0.05 compared to HRECs^OE-VEGF^ with the SCU glucose+CoCl_2_. (h) Western blot analysis of p-FAK, p-Src, and p-ERK in HRECs and HRECs^OE-VEGF^ treated with glucose+CoCl_2_+SCU.

To illuminate VEGF playing an important role on improving angiogenesis under diabetic conditions, we knocked down VEGF in HRECs (HRECs^kd-VEGF^) at the diabetic environment. The results indicated that silencing of VEGF expression in HRECs with siRNA ameliorated the diabetes-induced migration (Figures 5(a)–5(c)). In addition, the phosphorylation of ERK, Src, and FAK activated by glucose and CoCl_2_ was impeded as a result of VEGF knockdown (Figure 5(d)). These results demonstrated that VEGF was an important inducer of angiogenesis and the activation of p-ERK, p-Src, and p-FAK under high glucose and hypoxia. We next used VEGF-overexpressing HREC models (HRECs^OE-VEGF^) to investigate whether SCU decreased angiogenesis through regulating the expression of VEGF in the diabetic environment. HREC migration was increased by glucose and CoCl_2_. Importantly, overexpression of VEGF reduced the antiangiogenic effects of SCU in HRECs (Figures 5(e)–5(g)). Furthermore, VEGF, p-ERK, p-Src, and p-FAK were activated in HRECs under the diabetic environment. The treatment of SCU in HRECs attenuated the expression of VEGF, p-ERK, p-Src, and p-FAK, while this effect of SCU was faded by overexpressing of VEGF in HRECs. These results indicated that SCU could not directly regulate the activation of p-ERK, p-Src, and p-FAK, and it inactivated p-ERK, p-Src, and p-FAK by decreasing the expression of VEGF under high glucose and hypoxia condition.

### 3.5. General Features of the Rat Model of Type 2 Diabetes Mellitus (T2DM)

To test our hypothesis that oral administration of SCU could be used to inhibit angiogenesis in DR, we used a rat model of T2DM to determine the efficacy and mechanism of SCU. High blood glucose and decreased body weight are considered to be the most important symptoms of diabetes. The blood glucose levels and body weights of the experimental rats are shown in Tables 1 and 2. Diabetes was induced by streptozotocin (STZ) injection at week 8. Our results show that the mean blood glucose level was significantly elevated after STZ injection (at week 8), but no significant differences in blood glucose were measured between diabetic rats with or without SCU treatment (Table 1). Thus, a rat model of T2DM was successfully established by a high-fat diet (HFD) and injection of STZ. Table 1 also shows that intragastric administration of SCU could not decrease high blood glucose. Body weight in all groups increased until the administration of STZ at the end of week 8. After injection of STZ, the body weights of the rats in the DR group and the DR+SCU group began to drop. The DR+SCU group showed a greater body weight than the DR group, although the difference was not significant (*P* > 0.05) (Table 2). These results indicated that SCU did not affect the blood glucose levels and body weights of diabetic rats.

### 3.6. Color Doppler Imaging and Histologic Analysis of Vascular Changes in DR *In Vivo*

**Figure 6:**
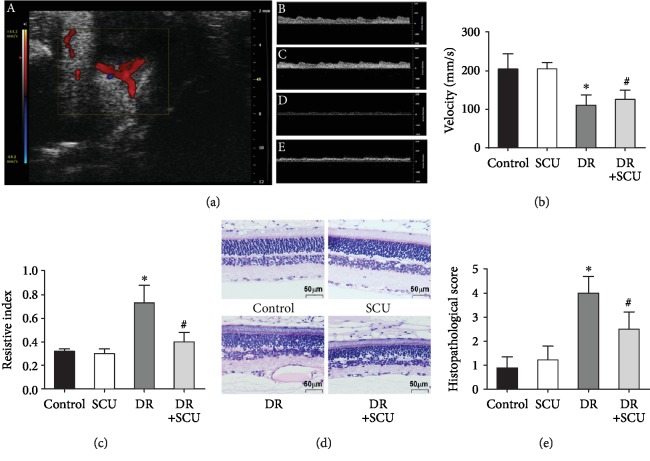
SCU improved diabetes-induced microcirculatory dysfunctions. (a) Color Doppler sonography analysis was performed in experimental animals. (A) Color Doppler ultrasound image from a DR+SCU rat retina showing localization of retinal blood vessel. (B–E) Spectral analysis of retinal blood flow rate in the control, SCU, DR, and DR+SCU groups, respectively. (b) Quantitative evaluation of velocity showed no significant difference between the DR group and the DR+SCU group (*P* > 0.05). (c) Quantitative evaluation of resistive index showed an increase of RI in the DR group (^∗^significantly different from the control group and the SCU group, *P* < 0.05) and a significant decrease in the DR+SCU group (^#^significantly different from the DR group, *P* < 0.05). There is no significant difference between the control group and the SCU group, *P* > 0.05. Histologic retinal section with hematoxylin-eosin staining showing retinal neovascularization on the retinal surface. (d) A cross section of the retina from the control group and the SCU group showing less neovascular nuclei on the retinal surface. A cross section of the retina from DR rats showing several neovascular nuclei on the retinal surface. A cross section of the retina from the SCU-treated DR group showing less retinal neovascularization. (e) Comparison of mean ± SD neovascular nucleus counts of the eyes included in the study. The values represent means calculated from four eyes per group with eight sections per eye (^∗^significantly different from the control group and the SCU group, *P* < 0.05; ^#^significantly different from the control group, the SCU group, and the DR group, *P* < 0.05; there is no significant difference between the control group and the SCU group, *P* > 0.05).

After two months of STZ injection, the resistive index (RI) of the retinal vessel, as assessed by color Doppler sonography, was significantly (^∗^*P* < 0.05) greater in the DR rats than in the control rats (Figure 6(c)), indicating abnormality of vasoconstriction in the retinas of DR rats. This increased RI was attenuated by oral administration of SCU (^#^*P* < 0.05 compared to the DR group). The retinal blood flow velocity, also measured by color Doppler sonography, was lower in the DR group than in the control group, but SCU treatment could not significantly attenuate the decrease (^#^*P* > 0.05 compared to the DR group, Figure 6(b)). These data revealed that SCU did not markedly influence the velocity of retinal vessel blood flow, but it did effectively ameliorate abnormal vasoconstriction. To confirm the protective effects of SCU in DR rats, histologic analysis was conducted to determine the levels of retinal neovascularization in the control group, the SCU group, the DR group, and the DR+SCU group. The number of neovascular nuclei on the retinal surface was determined in ten sections per eye to obtain a quantitative measure of the degree of retinal neovascularization for each eye. DR rats had markedly greater numbers of neovascular nuclei than the other groups (^∗^*P* < 0.05 compared to the control group and the SCU group) (Figure 6(d)). Interestingly, SCU treatment decreased the number of neovascular nuclei in DR rats (*P* < 0.05). These results are shown graphically in Figure 6(e). No signs of retinal structural damage were found in any group.

### 3.7. SCU Attenuated Diabetes-Induced VEGF Expression and Phosphorylation of ERK, FAK, and Src in the Retinas of DR Rats

**Figure 7:**
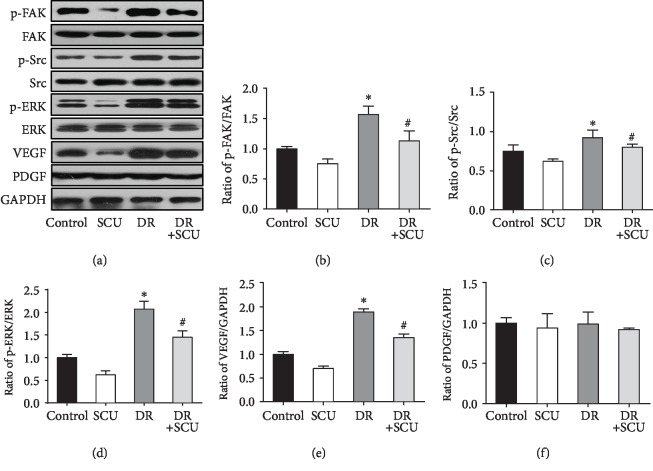
SCU downregulated the activation of p-ERK, p-FAK, p-Src, and VEGF but did not affect the expression of PEDF in the retina of DR rats. (a) Western blot analysis was used to determine the protein expression of VEGF, PEDF, FAK, p-FAK, Src, p-Src, ERK, and p-ERK in the retina of diabetic rats. GAPDH was used as loading control. Bar graphs in (b)–(f) present quantitative difference in expression of independent experiments. Data are presented as mean ± SD (*n* = 3); ^∗^significantly different from the control group and the SCU group, *P* < 0.05; ^#^significantly different from the control group, the SCU group, and the DR group, *P* < 0.05.

**Figure 8:**
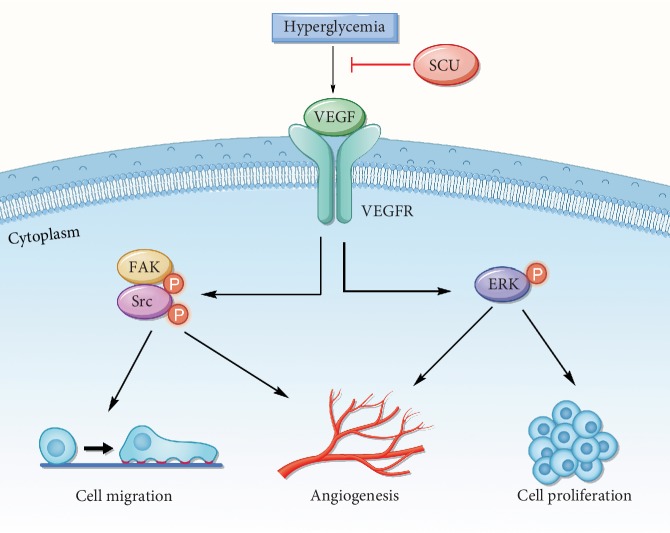
Schema: the antitubulogenic mechanism of SCU on diabetic retinopathy. SCU attenuates the retinal tubulogenesis induced by hyperglycemia and hypoxia, by decreasing the expression of VEGF and in turn suppressing the phosphorylation of FAK, Src, and ERK.

To confirm the antiangiogenic effects of SCU and its mechanism in DR rats, we investigated the retinal expression of VEGF, PEDF, p-ERK, p-FAK, and p-Src. Compared with that in the retinas of control rats, the protein expression of VEGF, p-ERK, p-Src, and p-FAK was significantly increased in the retinas of DR rats (Figures 7(a)–7(e)). Treatment of DR rats with SCU reduced VEGF production and further downregulated p-ERK, p-Src, and p-FAK expression. As expected, there was little change in the expression of PEDF in the DR group (Figures 7(a) and 7(f)). The *in vivo* model results, namely, oral administration of SCU in DR rats effectively decreased the crosstalk of the angiogenic pathway proteins VEGF, p-ERK, p-Src, and p-FAK in the retina, were consistent with the *in vitro* results. The mechanism is described in the schema (Figure 8).

## 4. Discussion

Diabetes mellitus (DM) can lead to dysfunctions in multiple organs and systems because of hyperglycemia and hypoxia. Insulin resistance, or reduced responsiveness of body tissues to insulin, is the hallmark of type 2 diabetes mellitus (T2DM). Insulin resistance always results in damage to small blood vessels, especially diabetic retinopathy (DR) [34–36]. DR is one of the most common diabetic microvascular complications and is a leading cause of blindness. Some previous studies have demonstrated that retinal cells in hyperglycemic and hypoxic environments exhibit high levels of VEGF [37], which is a key mediator of neovascularization in DR pathogenesis. VEGF has been proven to participate in responses to hyperglycemia and hypoxia during periods of inadequate blood circulation by controlling endothelial metabolism [38, 39]. Therefore, the development of antiangiogenic agents has become an attractive new strategy to treat DR. Our study firstly showed that SCU could decrease the angiogenesis in HRECs under the stimulated diabetes condition and it also alleviated the retinal microvascular dysfunction in DR rats by oral administration. Secondly, our study suggested that SCU exerted its antiangiogenic effects *in vitro* and *in vivo* by impeding the crosstalk of VEGF, p-ERK, p-FAK, and p-Src, which were activated by high glucose/hypoxia.

SCU has been extensively used in treating diabetes and its complications, as it can prevent vascular dysfunction [14, 15, 16]. In particular, it has also been considered as a potential therapeutic agent for DR [40]. Our study showed that SCU had antiangiogenic effects on protecting DR. In an *in vitro* study, we found that SCU seldom affected glycolipid metabolism in HRECs under the stimulated diabetes condition, but it could decrease the diabetes-induced migration and tube formation of HRECs, which proved that SCU inhibited HREC angiogenesis by other mechanisms instead of decreasing glycolipid metabolism. HRECs have been used as models to investigate the pathogenic mechanisms of DR because these cells are important for maintaining neovascularization [29, 41]. This result is consistent with our *in vivo* experiments. After administration of SCU, the blood glucose and body weight of T2DM rats showed few changes, but the retinal microvascular dysfunction was alleviated. Our previous study also indicated that SCU can attenuate diabetes-induced microcirculatory impairment of the testes by inhibiting oxidative stress, but not affecting glycolipid metabolism [21]. Accordingly, reduction of glycolipid metabolism was not the mechanism of SCU against diabetes dysfunctions.

The mechanisms of SCU against diabetes-induced angiogenesis have not been fully elucidated. Therefore, our study investigated the antiangiogenic mechanism of SCU under diabetic condition *in vitro* and in *vivo*. The signal pathway of ERK, Src, and FAK has been proven to engage in crosstalk to regulate cell angiogenesis. ERK is a member of the MAPK pathway, and activation of ERK by phosphatidylinositol 3-kinase has been connected with vascular angiogenesis [42]. Previous reports have suggested that inhibition of p-ERK reverses angiogenesis and slows the progression of vascular diseases. The antiangiogenic agent decreased VEGF and ERK at the same time to inhibit the endothelial migration and tube formation [43]. On the other hand, the cells move toward an angiogenic phenotype with the activation of p-ERK and VEGF [44].

Focal adhesion kinase (FAK) is a major nonreceptor tyrosine kinase activated after integrin-mediated adhesion to extracellular matrix (ECM) proteins. Autophosphorylation of FAK results in stimulation of a cell signaling cascade that ultimately activates the RAS/MAPK/ERK pathway [45]. As to FAK, members of the Src family of nonreceptor protein tyrosine kinases are also associated with focal adhesions and integrin signaling [46–48]. Src can bind to an autophosphorylation site on FAK and then phosphorylate other sites on FAK [49]. Following FAK activation, downstream signaling molecules can be activated, including molecules in the ERK pathway [50, 51]. Study indicated that the phosphorylation on Tyr1175 of VEGFR2 mediates activation of MAPK/ERK and has also been linked to activation of p-Src, which regulates vascular permeability and cell migration [52, 53]. Zhang et al. [54] reported that SKLB1002 exerts antiangiogenic effects by inhibiting the binding of VEGF and VEGFR, subsequently impeding the activity of p-ERK, p-FAK, and p-Src downstream. In the above studies, the crosstalk of VEGF, p-ERK, p-FAK, and p-Src has been strongly proven to promote angiogenesis. Thus, inhibition of the crosstalk of p-ERK, p-FAK, p-Src, and VEGF has been considered as an antiangiogenic strategy. Our results demonstrated that high glucose and hypoxia could induce the expression of VEGF and the subsequent phosphorylation of ERK, FAK, and Src, in HRECs, which related to angiogenesis of HRECs. Importantly, knockdown of VEGF in HRECs attenuated the phosphorylation of ERK, FAK, and Src induced by high glucose/hypoxia. These results proved that VEGF is an important mediator of the angiogenesis crosstalk induced by high glucose/hypoxia. Our study also indicated that SCU decreased the expression of VEGF, blocked the activation of p-ERK, p-FAK, and p-Src, and inhibited cellular angiogenesis in HRECs. However, in HRECs^OE-VEGF^, SCU lost its ability of inhibiting activation of p-ERK, p-FAK, and p-Src. Finally, we suggested that SCU exerts the antiangiogenic functions by downregulating VEGF and then impeding the crosstalk of VEGF/p-ERK/p-Src/p-FAK.

Pigment epithelium-derived factor (PEDF) is an antiangiogenic factor that has been proven to decrease the expression of VEGF in diabetes [55–57]. Our results indicated that SCU decreased the expression of VEGF not through regulating PEDF. Possibly, SCU also regulated other molecules to decrease angiogenesis, but in our study, we proved that SCU indeed decreased the diabetes-induced angiogenesis *in vivo* and *in vitro* and these effects are related to the downregulation of the crosstalk of VEGF, p-ERK, p-FAK, and p-Src. In the future, we will point out the clearer mechanism and pathway of SCU of DR.

Doppler sonography was used in our *in vivo* study to measure the retinal vessels, which is a noninvasive and sensitive technique that enables real-time recording in the chosen blood vessels. Although this technique has been used in human diabetic patients, it has not been widely used previously in diabetic animals. Our study showed ultrasound images of abnormal retinal vasoconstriction in diabetic rats by Doppler sonography. Abnormal retinal vasoconstriction has been proven to result in severe vitreous cavity hemorrhage or retinal detachment [58]. Dimitrova et al. used color Doppler ultrasound to provide evidence that RI is significantly increased in the posterior ciliary artery and central retinal artery of diabetic retinas [59]. In our study, RI was increased in the DR group, but it was attenuated in the DR+SCU group, showing that SCU effectively alleviated DR-induced vasoconstriction. Several studies have illustrated that increased vasoconstriction, a kind of vascular abnormality, occurs in many organs in diabetes [60]. It is possible for blood flow to increase or decrease based on the degree of vasoconstriction within a physiologic range. The increased RI was indicative of distal vasoconstriction, such as constriction at the precapillary or capillary level that resulted in decreased blood flow in the retinal capillary bed, though the exact degree of blood flow reduction was not measured. A previous study reported that abnormal vasoconstriction caused by hypoxia occurs along with increases in VEGF [61] and inhibitors of the VEGF pathway have been regarded as promising therapeutic agents for relieving vasoconstriction [62]. To our surprise, oral administration of SCU in diabetic rats not only decreased retinal angiogenesis but also corrected abnormal vasoconstriction by inhibiting the expression of VEGF. To achieve this objective of DR treatment through oral administration of SCU, many further studies are needed to develop SCU as a drug candidate. For example, careful pharmacokinetic studies will be necessary to determine the duration, distribution, and availability of SCU in ocular tissues.

Taken together, our results *in vitro* and *in vivo* showed that SCU potently and specifically inhibits DR neovascularization by inhibiting the expression of VEGF and the activation of the angiogenesis crosstalk involving p-ERK, p-FAK, and p-Src. Importantly, oral administration of SCU exerted antiangiogenic effects in the DR rat model, suggesting that oral administration could be used instead of intravitreal injection to prevent and treat DR in the future. Therefore, SCU is a promising candidate oral antiangiogenic agent for the reduction of retinal neovascularization in DR.

## Figures and Tables

**Table 1:** Effect of scutellarin on blood glucose in STZ-induced diabetic rats.

		Time		
	Groups	Week 8	Week 12	Week 16
Blood glucose (mmol/l)	Control	6.16 ± 1.22	6.01 ± 0.81	6.2 ± 0.44
	SCU	5.55 ± 0.83	5.68 ± 1.04	5.93 ± 1.54
	DR	5.33 ± 0.69	30.96 ± 1.78^∗^	29.98 ± 2.14^∗^
	DR+SCU	5.73 ± 0.54	29.16 ± 3.22^∗^	26.8 ± 2.65^∗^

^∗^Significantly different from the control group (*P* < 0.05). ^#^Significantly different from the diabetes group (*P* < 0.05). Values are expressed as mean ± SD.

**Table 2:** Effect of scutellarin on body weight in STZ-induced diabetic rats.

		Time			
	Groups	Week 4	Week 8	Week 12	Week 16
Body weight (g)	Control	252.33 ± 14.4	273.33 ± 24.59	309.16 ± 28.12	349.16 ± 21.63
	SCU	255.33 ± 12.16	287.5 ± 11.33	316.33 ± 15.02	340.66 ± 25.59
	DR	271.66 ± 6.28	316.5 ± 20.66	239.5 ± 23.08^∗^	240.13 ± 28.69^∗^
	DR+SCU	273.5 ± 16.64	322.66 ± 24.16	251.83 ± 26.28^∗^	243.83 ± 20.69^∗^^#^

^∗^Significantly different from the control group (*P* < 0.05). ^#^Significantly different from the diabetes group (*P* < 0.05). Values are expressed as mean ± SD.

## Data Availability

The data used to support the findings of this study are available from the corresponding authors upon request.

## Supplementary Materials

Supplementary Materials Chemical structure of scutellarin.

## References

[1] Bin Dhuban K., Kornete M., Mason E. S., Piccirillo C. A. (2014). Functional dynamics of Foxp3^+^ regulatory T cells in mice and humans. *Immunological Reviews*.

[2] Hautala N., Aikkila R., Korpelainen J. (2014). Marked reductions in visual impairment due to diabetic retinopathy achieved by efficient screening and timely treatment. *Acta Ophthalmologica*.

[3] Ibrahim A., el-meligy A., Lungu G. (2011). Curcumin induces apoptosis in a murine mammary gland adenocarcinoma cell line through the mitochondrial pathway. *European Journal of Pharmacology*.

[4] Miller J. W., Adamis A. P., Aiello L. P. (1997). Vascular endothelial growth factor in ocular neovascularization and proliferative diabetic retinopathy. *Diabetes / Metabolism Reviews*.

[5] Li Y., Busoy J. M., Zaman B. A. A. (2018). A novel model of persistent retinal neovascularization for the development of sustained anti-VEGF therapies. *Experimental Eye Research*.

[6] Xu Y., Lu X., Hu Y. (2018). Melatonin attenuated retinal neovascularization and neuroglial dysfunction by inhibition of HIF-1*α*-VEGF pathway in oxygen-induced retinopathy mice. *Journal of Pineal Research*.

[7] He F., Xia X., Wu X. F., Yu X. Q., Huang F. X. (2013). Diabetic retinopathy in predicting diabetic nephropathy in patients with type 2 diabetes and renal disease: a meta-analysis. *Diabetologia*.

[8] Banji D., Pinnapureddy J., Banji O. J. F., Saidulu A., Hayath M. S. (2011). Synergistic activity of curcumin with methotrexate in ameliorating Freund's Complete Adjuvant induced arthritis with reduced hepatotoxicity in experimental animals. *European Journal of Pharmacology*.

[9] Reeta K. H., Mehla J., Gupta Y. K. (2010). Curcumin ameliorates cognitive dysfunction and oxidative damage in phenobarbitone and carbamazepine administered rats. *European Journal of Pharmacology*.

[10] Yang B., Zhao Y. L., Yang X. (2013). Scutellarin-cyclodextrin conjugates: synthesis, characterization and anticancer activity. *Carbohydrate Polymers*.

[11] Ma G. Y., Cao Y. F., Hu C. M. (2014). Comparison of inhibition capability of scutellarein and scutellarin towards important liver UDP-glucuronosyltransferase (UGT) isoforms. *Phytotherapy Research*.

[12] Kheradpezhouh E., Panjehshahin M. R., Miri R. (2010). Curcumin protects rats against acetaminophen-induced hepatorenal damages and shows synergistic activity with N-acetyl cysteine. *European Journal of Pharmacology*.

[13] Hua W. F., Fu Y. S., Liao Y. J. (2010). Curcumin induces down-regulation of EZH2 expression through the MAPK pathway in MDA-MB-435 human breast cancer cells. *European Journal of Pharmacology*.

[14] Sompamit K., Kukongviriyapan U., Nakmareong S., Pannangpetch P., Kukongviriyapan V. (2009). Curcumin improves vascular function and alleviates oxidative stress in non-lethal lipopolysaccharide-induced endotoxaemia in mice. *European Journal of Pharmacology*.

[15] Kunwar A., Sandur S. K., Krishna M., Priyadarsini K. I. (2009). Curcumin mediates time and concentration dependent regulation of redox homeostasis leading to cytotoxicity in macrophage cells. *European Journal of Pharmacology*.

[16] Chen X. J., Cheng D. Y., Yang L., Xia X. Q., Guan J. (2006). Effect of breviscapine on fractalkine expression in chronic hypoxic rats. *Chinese Medical Journal*.

[17] Wang M., Zhang W. B., Zhu J. H., Fu G. S., Zhou B. Q. (2009). Breviscapine ameliorates hypertrophy of cardiomyocytes induced by high glucose in diabetic rats via the PKC signaling pathway. *Acta Pharmacologica Sinica*.

[18] Chen J., Zhao Y. H., Liu X. L. (2012). Effects of breviscapine on pulmonary inflammatory response and lung injury in children undergoing open heart surgery. *Journal of Asian Natural Products Research*.

[19] Xie W. X., Yue L. M., Song H. L. (2010). Protective effect of breviscapine on cardiac function in children after cardiopulmonary bypass undergoing open heart surgery. *Chinese Journal of Integrated Traditional & Western Medicine*.

[20] Cao W., Liu W., Wu T., Zhong D., Liu G. (2008). Dengzhanhua preparations for acute cerebral infarction. *Cochrane Database of Systematic Reviews*.

[21] Long L., Wang J., Lu X. (2015). Protective effects of scutellarin on type II diabetes mellitus-induced testicular damages related to reactive oxygen species/Bcl-2/Bax and reactive oxygen species/microcirculation/staving pathway in diabetic rat. *Journal of Diabetes Research*.

[22] Piret J. P., Mottet D., Raes M., Michiels C. (2002). CoCl_2_, a chemical inducer of hypoxia-inducible factor-1, and hypoxia reduce apoptotic cell death in hepatoma cell line HepG2. *Annals of the New York Academy of Sciences*.

[23] Chen R., Xu J., She Y. (2018). Necrostatin-1 protects C2C12 myotubes from CoCl2-induced hypoxia. *International Journal of Molecular Medicine*.

[24] Colwell D. R., Higgins J. A., Denyer G. S. (1996). Incorporation of 2-deoxy-d-glucose into glycogen. Implications for measurement of tissue-specific glucose uptake and utilisation. *The International Journal of Biochemistry & Cell Biology*.

[25] Kanazawa S., Fujiwara T., Matsuzaki S. (2010). bFGF regulates PI3-kinase-Rac1-JNK pathway and promotes fibroblast migration in wound healing. *PLoS One*.

[26] Weir G. C., Clore E. T., Zmachinski C. J., Bonner-Weir S. (1981). Islet secretion in a new experimental model for non-insulin-dependent diabetes. *Diabetes*.

[27] Weksler-Zangen S., Yagil C., Zangen D. H., Ornoy A., Jacob H. J., Yagil Y. (2001). The newly inbred cohen diabetic rat: a nonobese normolipidemic genetic model of diet-induced type 2 diabetes expressing sex differences. *Diabetes*.

[28] Deng D., Evans T., Mukherjee K., Downey D., Chakrabarti S. (1999). Diabetes-induced vascular dysfunction in the retina: role of endothelins. *Diabetologia*.

[29] Cervellati F., Cervellati C., Romani A. (2014). Hypoxia induces cell damage via oxidative stress in retinal epithelial cells. *Free Radical Research*.

[30] Wang Y., Tang Z., Xue R. (2012). Differential response to CoCl_2_-stimulated hypoxia on HIF-1*α*, VEGF, and MMP-2 expression in ligament cells. *Molecular and Cellular Biochemistry*.

[31] Sairam K., Hemalatha S., Kumar A. (2003). Evaluation of anti-diarrhoeal activity in seed extracts of *Mangifera indica*. *Journal of Ethnopharmacology*.

[32] Ye X., Ren H., Zhang M., Sun Z., Jiang A. C., Xu G. (2012). ERK1/2 signaling pathway in the release of VEGF from Müller cells in diabetes. *Investigative Ophthalmology & Visual Science*.

[33] Long L., Qiu H., Cai B. (2018). Hyperglycemia induced testicular damage in type 2 diabetes mellitus rats exhibiting microcirculation impairments associated with vascular endothelial growth factor decreased via PI3K/Akt pathway. *Oncotarget*.

[34] He X., Li M., Guo F., Xie D. (2012). Reduced VEGF signaling in corpus cavernosum of rat with alloxan induced type I diabetes mellitus. *Life Science Journal*.

[35] Gibbons C. H., Freeman R. (2015). Treatment-induced neuropathy of diabetes: an acute, iatrogenic complication of diabetes. *Brain*.

[36] Tzeng T. F., Liu W., Liou S. S., Hong T. Y., Liu I. M. (2016). Antioxidant-rich extract from plantaginis semen ameliorates diabetic retinal injury in a streptozotocin-induced diabetic rat model. *Nutrients*.

[37] Caires K. C., de Avila J., McLean D. J. (2009). Vascular endothelial growth factor regulates germ cell survival during establishment of spermatogenesis in the bovine testis. *Reproduction*.

[38] Qaum T., Xu Q., Joussen A. M. (2001). VEGF-initiated blood-retinal barrier breakdown in early diabetes. *Investigative Ophthalmology & Visual Science*.

[39] Ellis E. A., Guberski D. L., Somogyi-Mann M., Grant M. B. (2000). Increased H_2_O_2_, vascular endothelial growth factor and receptors in the retina of the BBZ/Wor diabetic rat. *Free Radical Biology & Medicine*.

[40] Gao R., Zhu B. H., Tang S. B., Wang J. F., Ren J. (2008). Scutellarein inhibits hypoxia- and moderately-high glucose-induced proliferation and VEGF expression in human retinal endothelial cells. *Acta Pharmacologica Sinica*.

[41] Economou M. A., Wu J., Vasilcanu D. (2008). Inhibition of VEGF secretion and experimental choroidal neovascularization by picropodophyllin (PPP), an inhibitor of the insulin-like growth factor-1 receptor. *Acta ophthalmologica*.

[42] Parra J. L., Buxade M., Proud C. G. (2005). Features of the catalytic domains and C termini of the MAPK signal-integrating kinases Mnk1 and Mnk2 determine their differing activities and regulatory properties. *The Journal of Biological Chemistry*.

[43] Chiu W. C., Lin J. Y., Lee T. S., You L. R., Chiang A. N. (2013). *β*
_2_-Glycoprotein I inhibits VEGF-induced endothelial cell growth and migration via suppressing phosphorylation of VEGFR2, ERK1/2, and Akt. *Molecular and Cellular Biochemistry*.

[44] Meadows K. N., Bryant P., Pumiglia K. (2001). Vascular endothelial growth factor induction of the angiogenic phenotype requires Ras activation. *The Journal of Biological Chemistry*.

[45] Sawai H., Okada Y., Funahashi H. (2005). Activation of focal adhesion kinase enhances the adhesion and invasion of pancreatic cancer cells via extracellular signal-regulated kinase-1/2 signaling pathway activation. *Molecular Cancer*.

[46] Playford M. P., Schaller M. D. (2004). The interplay between Src and integrins in normal and tumor biology. *Oncogene*.

[47] Bromann P. A., Korkaya H., Courtneidge S. A. (2004). The interplay between Src family kinases and receptor tyrosine kinases. *Oncogene*.

[48] Liu P. S., Jong T. H., Maa M. C., Leu T. H. (2010). The interplay between Eps8 and IRSp53 contributes to Src-mediated transformation. *Oncogene*.

[49] Caron-Lormier G., Berry H. (2005). Amplification and oscillations in the FAK/Src kinase system during integrin signaling. *Journal of Theoretical Biology*.

[50] Grethe S., Porn-Ares M. I. (2006). p38 MAPK regulates phosphorylation of Bad via PP2A-dependent suppression of the MEK1/2-ERK1/2 survival pathway in TNF-*α* induced endothelial apoptosis. *Cellular Signalling*.

[51] Puente L. G., He J. S., Ostergaard H. L. (2006). A novel PKC regulates ERK activation and degranulation of cytotoxic T lymphocytes: plasticity in PKC regulation of ERK. *European Journal of Immunology*.

[52] Takahashi T., Yamaguchi S., Chida K., Shibuya M. (2001). A single autophosphorylation site on KDR/Flk-1 is essential for VEGF-A-dependent activation of PLC-*γ* and DNA synthesis in vascular endothelial cells. *The EMBO Journal*.

[53] Eliceiri B. P., Puente X. S., Hood J. D. (2002). Src-mediated coupling of focal adhesion kinase to integrin *α*v*β*5 in vascular endothelial growth factor signaling. *The Journal of Cell Biology*.

[54] Zhang S., Cao Z., Tian H. (2011). SKLB1002, a novel potent inhibitor of VEGF receptor 2 signaling, inhibits angiogenesis and tumor growth *in vivo*. *Clinical Cancer Research*.

[55] Simo R., Carrasco E., Garcia-Ramirez M., Hernandez C. (2006). Angiogenic and antiangiogenic factors in proliferative diabetic retinopathy. *Current Diabetes Reviews*.

[56] Ogata N., Tombran-Tink J., Nishikawa M. (2001). Pigment epithelium-derived factor in the vitreous is low in diabetic retinopathy and high in rhegmatogenous retinal detachment. *American Journal of Ophthalmology*.

[57] Anukulthanakorn K., Parhar I. S., Jaroenporn S., Kitahashi T., Watanbe G., Malaivijitnond S. (2016). Neurotherapeutic effects of Pueraria mirifica extract in early- and late-stage cognitive impaired rats. *Phytotherapy Research*.

[58] Wang B., Li P. K., Ma J. X., Chen D. (2018). Therapeutic effects of a novel phenylphthalimide analog for corneal neovascularization and retinal vascular leakage. *Investigative Ophthalmology & Visual Science*.

[59] Dimitrova G., Kato S., Fukushima H., Yamashita H. (2009). Circulatory parameters in the retrobulbar central retinal artery and vein of patients with diabetes and medically treated systemic hypertension. *Graefe's Archive for Clinical and Experimental Ophthalmology*.

[60] King G. L., Brownlee M. (1996). The cellular and molecular mechanisms of diabetic complications. *Endocrinology and Metabolism Clinics of North America*.

[61] Singh M., Yadav S., Kumar M. (2018). The MAPK-activator protein-1 signaling regulates changes in lung tissue of rat exposed to hypobaric hypoxia. *Journal of Cellular Physiology*.

[62] Touyz R. M., Herrmann S. M. S., Herrmann J. (2018). Vascular toxicities with VEGF inhibitor therapies-focus on hypertension and arterial thrombotic events. *Journal of the American Society of Hypertension*.

