# Supplementary figures and images for: Scutellarin Prevents Angiogenesis in Diabetic Retinopathy by Downregulating VEGF/ERK/FAK/Src Pathway Signaling
Source: J Diabetes Res. 2019 Dec 28;2019:4875421. doi: 10.1155/2019/4875421 (PMC6949683; doi:10.1155/2019/4875421)

**Supplementary information 1.** Chemical structure of scutellarin


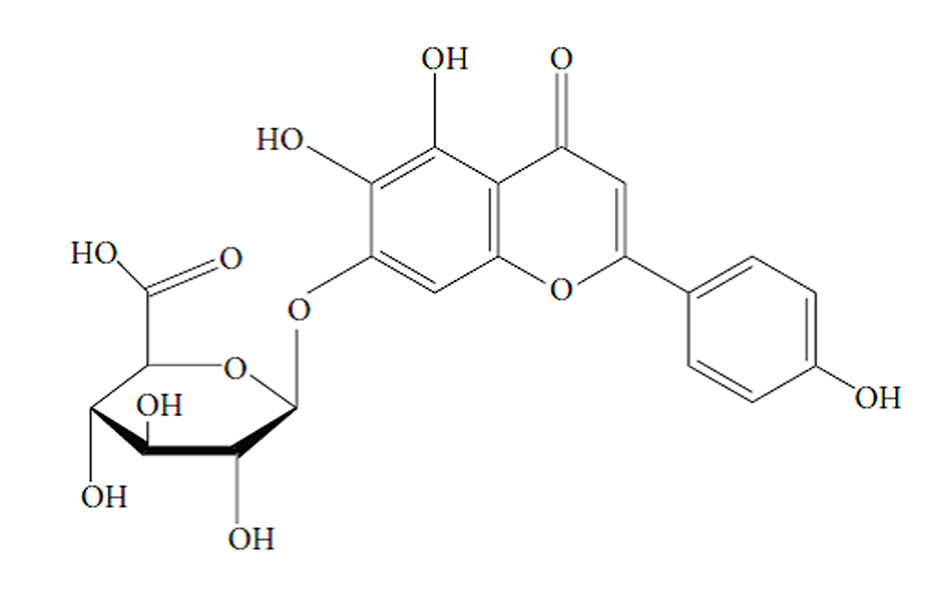

Supplement: Supplementary Materials — Chemical structure of scutellarin. [file 4875421.f1.docx]
